# Measuring the Reliability of Postural Sway Measurements for a Static Standing Task: The Effect of Age

**DOI:** 10.3389/fphys.2022.850707

**Published:** 2022-05-13

**Authors:** Pei-Yi Lo, Bo-Lin Su, Yu-Lin You, Chen-Wen Yen, Shih-Ting Wang, Lan-Yuen Guo

**Affiliations:** ^1^ Department of Sports Medicine, College of Medicine, Kaohsiung Medical University, Kaohsiung, Taiwan; ^2^ Department of Mechanical and Electro-mechanical Engineering, National Sun Yat-Sen University, Kaohsiung, Taiwan; ^3^ College of Medicine, Kaohsiung Medical University, Kaohsiung, Taiwan; ^4^ Department of Medical Research, Kaohsiung Medical University Hospital, Kaohsiung, Taiwan; ^5^ College of Health Sciences, Kaohsiung Medical University, Kaohsiung, Taiwan; ^6^ College of Humanities and Social Sciences, National Pingtung University of Science and Technology, Pintung, Taiwan

**Keywords:** age effect, balance ability, reliability, force plate, center of pressure

## Abstract

**Background:** A force plate is used to determine the ability to balance ability. However, only some medical centers or laboratories are equipped with force plates because they are costly so a low-cost force plate is required for home care or health care institutes. Few studies compare the reliability of postural sway measurements in terms of age. This study proposes a low-cost force plate to select reliable parameters to evaluate postural sway.

**Objectives:** To determine the intra-rater reliability of a novel force plate and the effect of age difference on the intra-rater test-retest reliability for the center of pressure (COP).

**Methods:** Forty participants were enrolled for this study: 20 youths and 20 older adults. Participants stood on a custom-made and low-cost force plate with eyes opened and eyes closed to measure COP-related parameters. The within-day test-retest reliability was measured at two sessions on the same day and the between-days reliability was measured on two different days. The COP-related parameters include the average velocity of COP, the average velocity in the antero-posterior and medio-lateral directions, the mean distance of COP and the mean distance in the antero-posterior and medio-lateral directions. An intra-class correlation coefficient test with one-way random model was performed to determine the reliability of different variables within-days and between-days. The results were presented in single measurement of intraclass correlation coefficient (ICC), the standard error of measurements, and the minimal detectable changes of each COP-related parameters.

**Results:** The novel low-cost force plate demonstrates excellent reliability in terms of the COP velocity related parameters for within- and between-day measurements. The ICC of COP distance related parameters were good to excellent reliability for between-day measurements (range: 0.43–0.84). Older adults demonstrated excellent reliability in terms of the mean distance for antero-posterior and the results were better than those for younger participants for the eyes-opened and eyes-closed conditions. The reliability in terms of the mean distance for medio-lateral was poor to good for older adults (range: 0.38–0.55), and excellent for younger participants.

**Conclusion:** The novel and low-cost force plate reliably measured balance and age affects the reliability of different COP variables, so the results of this study were pertinent to the selection of COP measures.

## 1 Introduction

Falling and the related problems afflict the older adults (Corriveau et al., 2001). For normal activities, posture control involves adjusting body’s direction and balance when standing (Lafond et al., 2004). Many reasons for falling have been proposed (Tinetti 1987). One of the most common causes is reduced ability to balance. Balance is the ability to control body coordination when moving or maintaining a loading posture (Sheldon 1963; Wolfson et al., 1986; Woollacott et al., 1986; Tracey et al., 2012; Huang and Yang 2019; Liu 2021).

Balance involves coordinating the transfer of the center of mass and the center of pressure (COP). For different postures and movements, the central nervous system uses inputs from vision, vestibular sense and proprioception to maintain balance. The deterioration of balance in the older adults can cause falls (Lord and Dayhew 2001). the ability to balance decreases with age because sensory inputs are changed and older adults who exhibit poor postural control demonstrate greater muscle co-activation to compensate for a decline in proprioception (Manchester et al., 1989; Nagai et al., 2011). The deterioration of proprioception can increase reliance on feedforward during dynamic tasks (Piirainen et al., 2013). Balance intervention is used to improve proprioception and postural control (Ross and Guskiewicz 2006; Wortmann and Docherty 2013; Nam et al., 2018). The ability to control posture is measured by measuring the center of pressure while standing on a force plate. Balance can be measured subjectively and objectively. Subjective methods involve a questionnaire assessment, that is, limited to a specific age or personal recognition disorder. Objective methods measure the COP excursion, the postural sway and the distribution of loading (Berg et al., 1989; Prieto et al., 1993; Kairy et al., 2003; Anker et al., 2008; Blum and Korner-Bitensky 2008; Mercer et al., 2009). A force plate is used to evaluate the balance ability by calculating COP-related parameters, such as the excursion velocity or the displacement, to give information about posture control (Palmieri et al., 2002). Force plates are expensive because the force sensors must measure the three-dimensional orientation of the force. They are too expensive for home care or community care settings so this study used a custom-made novel and low-cost force plate that uses four force sensors, but simplifies the measurement to a one-dimensional orientation force, so it is significantly cheaper than current options (Su et al., 2015; Hong et al., 2016; Hong et al., 2017).

Balance ability can be evaluated by observing the COP excursion and COP-related parameters, such as excursion velocity. However, the COP-related parameters that are obtained from the force plate must be reliable. To reliably measure the ability to balance, the reliability of the proposed force plate must be determined prior to its use to measure the ability to balance. Reliability is a measurement of the ability to achieve similar results for different measurement times for stable individuals (Guyatt et al., 1992). A reliable force plate is an essential element of any system to measure the ability to balance and reliable parameters must be used to prevent clinical failures. The measurement of the effectiveness of any method of balance intervention requires reliable parameters (Corriveau et al., 2001). The use of the force plate to measure COP is a validated and reliable method to evaluate balance performance (Li et al., 2016). The reliability of COP measures has been investigated in the previous studies (Carpenter et al., 2001; Bauer et al., 2008; Swanenburg et al., 2008; Pinsault and Vuillerme 2009; Moghadam et al., 2011; Da Silva et al., 2013; Li et al., 2016; Levy et al., 2018). The reliabilities of average COP velocity while quiet standing on the rigid and foam surface with eyes open and eyes close were ranged from 0.82 to 0.93 (Moghadam et al., 2011). In addition, older adults who sustained at least one fall within 1 year demonstrated lower reliabilities of COP-related parameters, such as, sway distance in anteroposterior (AP) and mediolateral (ML) direction than older adults who did not have fall experience within one year (Swanenburg et al., 2008). In previous study, older adults demonstrated greater reliability of average COP velocity in AP and ML direction compared to young adults (Lin et al., 2008). In addition, the reliability studies used intra-class correlation coefficient (ICC) to present relative reliability or absolute reliability, such as standard error of measurement (SEM) to test the reproducibility (Weir 2005). The COP velocity related parameters demonstrated good to excellent test retest reliability, and, the sway path distance exhibited good reliability in both eyes open and eye close conditions (Golriz et al., 2012; Hébert-Losier and Murray 2020). Hence the COP velocity and COP path distance related parameters are appropriate variables to evaluate balance ability.

Some studies showed that the mean distance of COP for older adults was greater than the value for young subjects for a balance test (Prieto et al., 1996; Slobounov et al., 2006). Many studies also identified significantly greater postural sway in the older population than in younger cohorts (Prieto et al., 1996; Slobounov et al., 2006). The effect of age on the reliability of postural sway measurements determines the variables that are used to evaluate postural sway and the effectiveness of any intervention. However, very few studies measure the reliability of COP-related parameters to determine which variables can be used to measure the ability to balance for different age groups.

This study determines the reliability of the novel and low-cost force plate for young participants and then for an older adult group to determine whether there are differences in reliability for different age groups. This study hypothesized that the novel and low-cost force plate exhibits sufficient within- and between-day reliability for use to evaluate the ability to balance by measuring COP-related parameters. It is also hypothesized that age affects the reliability of COP measurements.

## 2 Materials and Methods

### 2.1 Participants

A G*Power 3.1.9.7 program was used to calculate the sample size of the present study. The sample size was calculated according to the study design and the previous study (Fleiss 1986; Fritz et al., 2012). At least 28 participants were needed to achieve 80% statistical power with an alpha level of 0.05 for repeated measurement study design. The correlation among measurements were set at 0.80 (high reliability) with a moderate effect size (Cohen’s d equals to 0.5) (Fritz et al., 2012).

The sampling methods of this study was convenient sampling. Forty subjects (20 youths, average age: 20.1 ± 1.3 years, and 20 older adults, average age: 68.7 ± 2.9 years) participated in this study. The basic profiles are shown below. Subjects were 18–25 years old or 65–75 years old. Subjects with lower limb neuromuscular injuries (e.g.: polio, stroke), musculoskeletal injuries (e.g.: fractures) or pain in the lower limbs were excluded. This study is approved by the Institutional Review Board of Kaohsiung Medical University Hospital [Approval number: KMUHIRB-2012-08-07(I)].

### 2.2 Procedures

**FIGURE 1:**
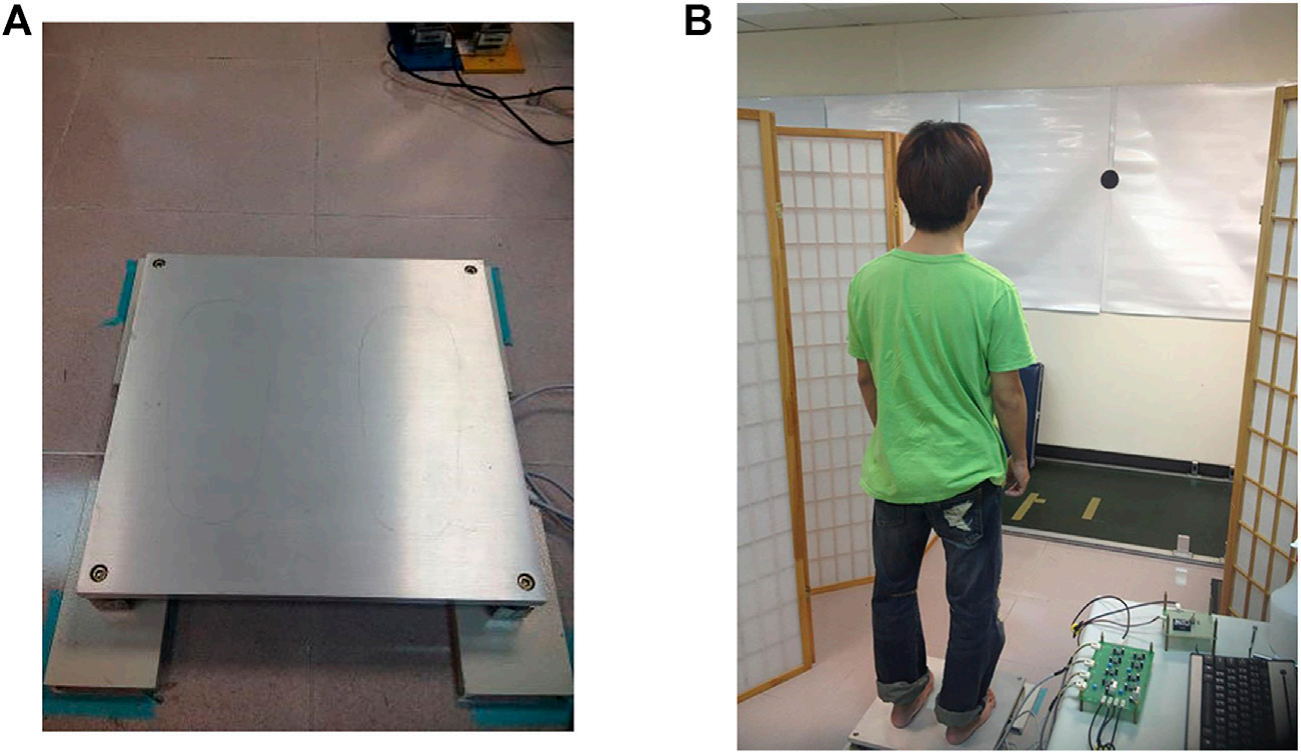
**(A)** The proposed low-cost force plate and **(B)** The experimental evironment.

This study included one investigator (rater) who had 2 years experiences in biomechanics investigation perform COP measures for all participants. Participants’ characteristics were determined before the test and questions were asked about medical records. Subjects then stood on a custom-made force plate (Figure 1A) to measure the height of their eyes from the ground. A mark was made 2 m from this height and the subject looked directly at the for the test. The mark was a circle with a diameter of 10 cm. In order to control across groups, participants were requested to stand on the center of force plate where there were feet-like marker. A previous study, the average step width of 18 healthy young (aged 27.7 years) participants was found to be 9.5 cm with 1.8 cm standard deviation (SD) and the average step width of 12 older adults was 10.4 cm with 3.4 cm SD (Owings and Grabiner 2004). Hence, the stance width of the present study was about 12 cm, which was measured by the distance of the heel between feet and the distance of the first metatarsal between feet.

A custom-made force plate contained 4 force sensors on the corner of the force plate same as the commercial force plate. However, the 4 force sensors installed in this custom-made force plate are single-axis. The experimental system used the graphical programming environment NI LabVIEW (National Instrument, Austin, TX, United States) for performing system control, signal processing, and graphical user interface (GUI) functions. Similar to a commercially available force platform, the force platform used in this work is a rectangular plate with force transducers located at its four corners. The size of the platform is 40 cm by 40 cm. Our previous work have carefully compared this force platform and a commercial force platform (Kistler 9286AA) to verify comparable repeatability and accuracy (Hong et al., 2016). After amplification, analog voltage signals obtained by the load cells of the force platform are converted to digital signals 24-bit DAQ (data acquisition) card (NI 9234). The digitized force signals were sent to a PC using a USB chassis (NI cDAQ-9174). The sampling frequency was set to 512 Hz.

The single-axis force sensor can be used to measure the balance performance while quiet standing because of that the vertical ground reaction force (GRF) is much larger than the anteroposterior (AP) and mediolateral (ML) GRFs. Hence, those GRFs can be considered negligible in computing the center of pressure while quiet standing task (Duarte and Freitas 2010; Huang et al., 2013; Bartlett et al., 2014). That’s why the balance board of Nintendo Wii can be a valid and reliable tool for evaluating the standing balance ability (Park and Lee 2014). The calculation of the COP in AP and ML with single-axis force sensor can be referred to the our previous study (Hong et al., 2016). Concluded above, the single axis force plate can be used to evaluate the balance performance while quiet standing.

A rater who had 2 years experiences in biomechanics investigation conducted the reliability evaluations. The protocol involved standing on two legs with eyes opened and closed three times for 40 s, with 1 min rest between each measurement session. During the examination, participants looked directly at the 2 m mark and stood on the marked spot on the force plate, with both arms naturally placed beside the thighs. Subjects refrained from deep breathing to minimize body sway and sound from outside the test environment was minimized (Figure 1B).

The reliability of the custom-made force plate was tested using within-day test-retests and between-day test-retests. Participants underwent the COP measures in the same laboratory environment, by the same investigator. The within day reliability test-retest were performed for two sessions on the same day by a break of 5 min and the between days reliability test-retest were performed for four different sessions on two different days. Participants underwent the same procedures and protocols on next day.

### 2.3 Data Analysis

The calculation of the COP in AP and ML with single-axis force sensor can be referred to the our previous study (Hong et al., 2016). This study used the data for 40 s and the first 5 s and the last 5 s of data were removed. The remaining 30 s was analyzed in terms of average velocity (V), medio-lateral average velocity (V-ML), antero-posterior average velocity (V-AP), mean distance (MD), medio-lateral mean distance (MD-ML) and antero-posterior mean distance (MD-AP).

There are 4 steps to calculate the mean distance including the mean COP in anteroposterior (AP) and in mediolateral (ML) directions, the AP and ML time series relative to the mean COP, the resultant distance time series and the mean distance (Prieto et al., 1996; Quijoux et al., 2021). First of all, the mean COP can be calculated as the averaged COP trajectory in AP and ML directions. The COP trajectory in AP and ML directions were noted as AP_0_ and ML_0_, then, the mean COP in AP and ML directions would be:

$$\begin{array}{l} \text{The}\;{\text{mean COP}}_{\text{AP}}=\frac{1}{N}\sum_{n=1}^{N}{\text{AP}}_{0},\;n\mathrm{=1,2,3,\ldots Ν}.\\ \text{The}\;{\text{mean COP}}_{\text{ML}}=\frac{1}{N}\sum_{n=1}^{N}{\text{ML}}_{0},\;n\mathrm{=1,2,3,\ldots Ν}. \end{array}$$ (1)

After that, the AP and ML time series were referenced to the mean COP can be calculated as following equations:

$$\begin{array}{l} \mathrm{AP}\left(n\right)=\sum_{n\mathrm{=1}}^{N}AP_{0}\left(n\right)\mathrm{-CO}P_{\mathrm{AP}}\\ \mathrm{ML}\left(n\right)=\sum_{n\mathrm{=1}}^{N}ML_{0}\left(n\right)\mathrm{-CO}P_{\mathrm{ML}} \end{array}$$ (2)

Resultant distance (RD) time series represented a distance between the mean COP and the COP in AP_0_ and ML_0_ time series as follows:

RD(n)=∑n=1N[AP(n)2+ML(n)2]12 (3)

Finally, the mean distance is the mean of the RD time series and that can be calculated as Eq. 4.

$$\mathrm{The}\;\mathrm{mean}\;\mathrm{distance}\left(\mathrm{mm}\right)=\frac{1}{Ν}\sum_{n\mathrm{=1}}^{N}\mathrm{RD}\left(n\right)$$ (4)

The mean velocity was calculated by averaging the total excursion over time (Prieto et al., 1996; Quijoux et al., 2021).

The total excursion(mm)=∑n=1N{[AP(n+1)−AP(n)]2+[ML(n+1)−ML(n)]2}12 (5)

$$\mathrm{The}\;\mathrm{mean}\;\mathrm{velocity}\left(mm/s\right)=\frac{Total\;excursion}{\text{Total time}}$$ (6)

### 2.4 Statistical Analysis

SPSS software version 20.0 (SPSS Inc. Chicago, IL) was used for statistical analysis. An intra-class correlation coefficient (ICC) with one-way random model was performed to determine the reliability of different variables within-days and between-days. The results were presented in single measurement of ICC, SEM and minimal detectable changes of COP-related parameters. An ICC value of greater than 0.75 represents excellent reliability, a value of between 0.4 and 0.75 represents fair or good reliability and a value of less than 0.4 represents poor reliability (Fleiss 1986). A 95% confidence interval (CI) is used to measure the precision of the estimate for each ICC value. The α level is 0.05. The SEM represented the variance extent between measurements, and, the smaller SEM, the greater reproducibility, the calculation of SEM of COP-related parameters was as follows: the standard deviation * (1-ICC coefficient)^1/2^, in addition, the minimal detectable changes of the COP-related parameters were also calculated.

## 3 Results

### 3.1 The Reliability of the Novel Force Plate

In terms of the within-day reliability measurement, the results for V (ICC: 0.91 and 0.84 for eyes open and eyes close conditions, respectively), V-ML (ICC: 0.87 and 0.87 for eyes open and eyes close conditions, respectively), V-AP (ICC: 0.97 and 0.84 for eyes open and eyes close conditions, respectively), MD (ICC: 0.56 and 0.75 for eyes open and eyes close conditions, respectively), and, MD-ML (ICC: 0.57 and 0.72 for eyes open and eyes close conditions, respectively) demonstrated good to excellent reliability for eyes-opened and eyes-closed tests (Table 1). The results for MD-AP showed poor reliability (ICC: 0.37 with SEM 0.91 and 0.60 with SEM 0.84 for eyes open and eyes close conditions, respectively) (Table 1).

**TABLE 1:** Within-day reliability for different COP variables in young participants.

		Session 1	Session 2	SEM	MDC	ICC (95%CI)
		Mean ± SD	Mean ± SD			
Average velocity (mm/s)	EO	9.96 ± 3.26	9.92 ± 2.85	0.81	2.25	0.93* (0.84,0.97)
	EC	12.01 ± 4.00	11.19 ± 3.62	1.53	4.23	0.84* (0.65,0.93)
Medio-lateral average velocity (mm/s)	EO	7.63 ± 3.02	7.22 ± 2.56	1.01	2.80	0.87* (0.70,0.95)
	EC	7.32 ± 3.02	6.76 ± 2.51	1.00	2.78	0.87* (0.70,0.94)
Antero-posterior average velocity (mm/s)	EO	11.78 ± 3.79	12.03 ± 3.69	0.65	1.80	0.97* (0.93,0.99)
	EC	15.00 ± 4.86	14.08 ± 4.81	1.93	5.36	0.84* (0.65,0.93)
Mean distance (mm)	EO	7.06 ± 1.68	7.48 ± 1.90	1.19	3.30	0.56 (0.17,0.80)
	EC	7.10 ± 2.13	7.09 ± 2.04	1.04	2.89	0.75* (0.48,0.90)
Medio-lateral mean distance (mm)	EO	3.51 ± 1.28	3.56 ± 1.51	1.11	3.08	0.57 (0.19,0.80)
	EC	2.91 ± 1.36	3.04 ± 1.37	0.86	2.39	0.72 (0.52,0.90)
Antero-posterior mean distance (mm)	EO	5.41 ± 1.28	5.77 ± 1.49	0.91	2.52	0.37 (−0.07,0.69)
	EC	5.85 ± 1.68	5.78 ± 1.48	0.84	2.32	0.60 (0.23,0.82)

*ICC, value > 0.75: excellent reliability.

EO, Eyes opened; EC, Eyes closed; ICC, Intraclass coefficient.

In terms of between-days reliability, the results for V, V-ML, and V-AP for the COP while standing with eyes-opened and eyes-closed demonstrated excellent reliability (ICCs for the eyes-opened test, with respective values of: 0.90, 0.89, and 0.89. ICC values for the eyes-closed are 0.91, 0.92, and 0.85, respectively. The MD, MD-ML, and MD-AP results demonstrate good to excellent reliability for the eyes-opened and eyes-closed tests (Table 2).

**TABLE 2:** Between-days reliability for different COP variables in young participants.

Between days		Day 1	Day 2	SEM	MDC	ICCs (95%CI)
		Mean ± SD	Mean ± SD			
Average velocity (V) (mm/s)	EO	9.94 ± 3.01	9.83 ± 2.23	0.42	1.18	0.90* (0.74,0.96)
	EC	11.60 ± 3.68	11.15 ± 2.96	0.68	1.88	0.91* (0.76,0.96)
Medio-lateral average velocity (mm/s)	EO	7.42 ± 2.71	7.53 ± 2.20	0.35	0.97	0.89* (0.73,0.96)
	EC	7.07 ± 2.71	7.12 ± 2.15	0.67	1.86	0.92* (0.79,0.97)
Antero-posterior average velocity (mm/s)	EO	11.93 ± 3.69	11.67 ± 2.66	0.32	0.89	0.89* (0.71,0.96)
	EC	14.54 ± 4.66	13.93 ± 3.74	0.45	1.24	0.85* (0.62,0.94)
Mean distance (mm)	EO	7.27 ± 1.59	7.15 ± 1.75	0.75	2.07	0.71 (0.26,0.89)
	EC	7.10 ± 1.94	7.42 ± 2.21	1.40	3.89	0.64 (0.10,0.86)
Medio-lateral mean distance (mm)	EO	3.54 ± 1.23	3.54 ± 1.31	0.48	1.32	0.61 (0.02,0.85)
	EC	2.98 ± 1.28	3.10 ± 1.25	0.80	2.22	0.50 (-0.26,0.80)
Antero-posterior mean distance (mm)	EO	5.59 ± 1.15	5.44 ± 1.30	0.63	1.74	0.81* (0.52,0.93)
	EC	5.82 ± 1.41	6.09 ± 1.74	1.15	3.20	0.80* (0.50,0.92)

*ICC, value > 0.75: excellent reliability.

EO, Eyes opened; EC, Eyes closed; ICC, Intraclass correlation coefficient; CI, Confidence interval.

### 3.2 The Reliability of Balance Ability Measurements for the Young and Older Adults

The reliability of the results for COP velocity, the V-AP and the V-ML for young participants and the older adults group demonstrate excellent reliability for the eyes-opened and eyes-closed test (Table 3). The reliability of the results for MD, MD-ML, and MD-AP for young participants demonstrated good to excellent reliability for the eyes-opened and eyes-closed tests.

**TABLE 3:** Between-days reliability for different COP variables for younger and older participants.

Tests	Youth		Older adults	
Parameters	EO	EC	EO	EC
Average velocity				
ICC	0.90*	0.91*	0.95*	0.98*
95%CI	0.74–0.96	0.76–0.96	0.88–0.98	0.95–0.99
SEM	0.42	0.68	0.49	0.49
MDC	1.18	1.88	1.36	1.36
Average velocity in AP				
ICC	0.89*	0.92*	0.96*	0.98*
95%CI	0.73–0.96	0.79–0.97	0.89–0.98	0.96–0.99
SEM	0.32	0.45	0.40	0.48
MDC	0.89	1.24	1.10	1.33
Average velocity in ML				
ICC	0.89*	0.85*	0.92*	0.94*
95%CI	0.71–0.96	0.62–0.94	0.80–0.97	0.84–0.98
SEM	0.35	0.67	0.23	0.21
MDC	0.97	1.86	0.65	0.59
Mean distance				
ICC	0.71	0.64	0.83*	0.91*
95%CI	0.26–0.89	0.10–0.86	0.58–0.93	0.77–0.96
SEM	0.75	1.40	0.94	1.08
MDC	2.07	3.89	2.59	2.99
Mean distance in AP				
ICC	0.61	0.50	0.93*	0.92*
95%CI	0.02–0.85	−0.26–0.80	0.83–0.97	0.80–0.97
SEM	0.63	1.15	0.54	0.99
MDC	1.74	3.20	1.51	2.75
Mean distance in ML				
ICC	0.81*	0.80*	0.38	0.55
95%CI	0.52–0.93	0.50–0.92	−0.56–0.76	−0.13–0.82
SEM	0.48	0.80	0.67	0.60
MDC	1.32	2.22	1.86	1.66

*ICC, value > 0.75: excellent reliability.

EO, Eyes opened; EC, Eyes closed; ICC, Intraclass correlation coefficient; CI, Confidence interval

For older adults, the results for MD and MD-AP demonstrated excellent reliability, which was higher than the results for young participants for the eyes-opened and eyes-closed tests. The reliability of the results for MD-ML for older adults was poor (for the eyes-opened tests) to good (for the eyes-closed tests), but younger participants demonstrated excellent reliability in terms of this parameter (Table 3). In the comparisons of the COP measures between age groups. Younger participants demonstrated smaller SEM in average velocity in AP direction and average distance in ML direction than that in the older adult group (Table 3). However, the older adult group demonstrated smaller SEM in the remaining parameters than that in the younger participant group (Table 3). The MDC values of COP-related parameters in younger participant group ranged from 0.67 to 1.36 for eyes-opened test, while, the MDC values in older adult group ranged from 0.51 to 1.36 for eyes-opened test. Besides, for eyes-closed test, the MDC values in younger participant group ranged from 0.78 to 1.88, and, values in older adult group ranged from 0.59 to 1.36 (Table 3).

## 4 Discussion

The proposed low-cost force plate can be used in clinics, for home care and in health care institutes because it is cheaper than a commercial force plate and is reliable equipment to measure the ability to balance. This is the first study to determine the test-retest reliability for a low-cost force plate for a protocol to measure the ability to balance and the first to determine the test-retest reliability of parameters to measure the ability to balance for different age groups, in order to determine the variables, measure the ability to balance for different age groups using this low-cost force plate.

Most of the results for within-day test-retest reliability in terms of COP-related parameters that were measured using the proposed low-cost force plate indicated good to excellent reliability and the between-day test-retest results demonstrated good to excellent reliability. Age affects the reliability of the MD-AP and MD-ML parameters. These results support the hypotheses for this study.

### 4.1 The Reliability of the Novel and Low-Cost Force Plate

In the current study, most of the COP-related parameters that were measured for static standing on two legs demonstrated good to excellent reliability for all except the MD-AP parameter for the within-day test-retest measurements. All of the COP-related parameters that are measured for this study demonstrate good to excellent between-day reliability.

The results for velocity for eyes-opened and eyes-closed tests demonstrated excellent between-day reliability, which was similar to the results of previous studies (Swanenburg et al., 2008; Moghadam et al., 2011). The between-day reliability for average velocity, V-AP and MD-AP for eyes-opened and eyes-closed test for older adults in the present study were excellent. The ICCs values were also higher than those for a previous study involving a balance evaluation protocol using a commercial force plate (AMTI, Watertown, MA, United States) for older adults (Swanenburg et al., 2008). In Lin et al. study, the SEM of mean velocity in AP and ML directions for between-day reliability measurement while performing quiet standing with eyes-closed were 1.2 and 2.1 (mm/s) in younger participants and 2.4 and 2.9 (mm/s) in older adults (Lin et al., 2008), which were greater than the present study at the same task condition (0.45 and 0.67 mm/s for mean velocity in AP and ML directions, respectively, in younger participants and 0.48 and 0.21 mm/s in AP and ML directions, respectively, in older adults). These results indicated that reproducibility of COP measures in those parameters were better by using the custom-made force plate than using commercial force plate (Lin et al., 2008). The SEM of average velocity in AP direction while young adults performing standing still for 10 repeated trials measured by the commercial force plate (Equi+, model PF01, Aix les Bains, France) in the previous study (Pinsault and Vuillerme 2009) was greater than the present study, moreover, the average velocity of older adults while performing quiet standing with eye-closed in the present study were smaller than that in the previous study (Swanenburg et al., 2008), which measured the balance protocol for 4 trials in one session while standing quietly with or without vision by using a commercial force plate with 50 Hz sampling rate (AMTI, United States) in older adults (Swanenburg et al., 2008). The MDC values of average velocity in AP and ML directions in both young (MDC for average velocity in AP: 1.24; ML: 1.86 mm/s) and older adults (MDC for average velocity in AP: 1.33; ML: 0.59 mm/s) of the present study were smaller than that in previous studies which used commercial force plate to measure COP of young (MDC for average velocity in AP: 3.33; ML: 5.82 mm/s) and older (MDC for average velocity in AP: 6.65; ML: 8.04 mm/s) adults (Lin et al., 2008).

The ICC results also demonstrated good reliability (ICCs: 0.71 for eyes-opened and 0.73 for eyes-closed tests) for healthy participants who did not have fall experience (Swanenburg et al., 2008). The ICC values for MD-ML were higher than the ICC values for MD-AP for within- and between-days for this study. Previous studies reported that the MD-ML can be used to determine whether the subject experiences falling (Bergland and Wyller 2004). These results showed that the proposed low-cost force plate demonstrates sufficient within- and between-day reliability (good to excellent) to be used to measure the ability to balance by measuring COP-related parameters. Some factors might influence the results of reliability measurements were reported (Hébert-Losier and Murray 2020). In the present study, we requested participants to look at a 10 cm diameter which was 2 m mark away from force plate, this might result in a more stable and constant measurement condition while performing eyes-opened test, because of that the head movement might affect the magnitude of sway. However, in the previous studies, they did not report whether participants have target to look at or not (Bauer et al., 2008; Lin et al., 2008; Swanenburg et al., 2008; Pinsault and Vuillerme 2009; Li et al., 2016).

### 4.2 The Effect of Age on the Reliability of Measurements of the Ability to Balance

Both groups for this study demonstrated excellent between-day reliability in terms of average COP velocity, V-AP and V-ML for the eyes-opened and eyes-closed test. The ICC values for V-AP, V-ML, and MD for the older adult group were higher than those for the younger group for this study. These findings were similar to those of a previous study, which also demonstrated excellent reliability for V-AP and V-ML and higher reliability in terms of these parameters for the older adults group than the younger group while performing upright and quiet standing with eyes-closed condition for three trials in each condition, as measured using a commercial force plate with 100 Hz sampling rate (AMTI OR6-7 series, Watertown, MA, United States) (Lin et al., 2008).

In the present study, for the eyes-opened and eyes-closed tests, only the older adult group demonstrated excellent reliability for the MD and MD-AP. The younger group demonstrated good reliability in terms of these parameters. Young participants demonstrated excellent reliability for MD-ML but the older adults group demonstrated poor to good reliability. This may be because movement for control in the AP direction is more accurate relative to the ML direction (Amoud et al., 2007), so there was a greater in the inter-session variation, which increases the ICC value. older adults who have a high risk of falling demonstrate a lower MD-AP value than young individuals and older adults who have a low risk of falling (Norris et al., 2005). Besides, older adults in this study demonstrated higher reliability for MD-AP than MD-ML. The decrease in the ability to balance with aging occurs primarily in the mediolateral direction (Day et al., 1993), so older adults may rely on vision to compensate for a decrease in balance in terms of lateral stability. Without vision, the variability increases between trials and the ICC values decreased, especially in the mediolateral direction (Day et al., 1993).

There were age-related differences in COP-measurements and that can be detected by the proposed low-cost force plate and the results are similar trends to those of previous studies that use commercial force plates. The older adults demonstrated better ICCs in average velocity in ML direction, and mean distance in AP direction with smaller SEM compared to younger participants. These results provided suggestions to choose reliable COP-related parameters to evaluate balance ability in older adults.

### 4.3 Study Limitations

Participants in the current study were healthy because the reliability evaluation must use stable individuals (Guyatt et al., 1992) to determine the reliability of equipment and the reliability of parameters to determine the effect of age. Other populations, such as individuals with experience of falling, must be studied to identify the aging effects on the reliability of parameters.

To measure the ability to balance, the present study requested participant to stand on two legs because standing on a single leg is dangerous for the older adults. Standing on one leg can also cause instability so there is greater variability in the studies. To determine the effect of age on COP-related parameters to measure the ability to balance, this study required participants to stand on two legs. However, a problem with balance may cause older adults to fall while walking or when obstacles are encountered (Chou et al., 2001), so these functional movements must be studied further.

## 5 Conclusion

The results for within- and between-day reliability indicate that the proposed low-cost force plate is a reliable tool for COP measurements for a static standing task with both eyes closed and both eyes opened, to determine the ability to balance.

Older adults demonstrated excellent test-retest reliability for MD-AP but young subjects demonstrate excellent reliability for MD-ML. This study provided suggestions for the selection of reliable COP-related parameters for a static standing task to measure the ability to balance for different age groups.

## Data Availability

The original contributions presented in the study are included in the article/Supplementary Material, further inquiries can be directed to the corresponding author.
