# Supplementary material for: Measuring the Reliability of Postural Sway Measurements for a Static Standing Task: The Effect of Age
Source: Front Physiol. 2022 May 13;13:850707. doi: 10.3389/fphys.2022.850707 (PMC9138610; doi:10.3389/fphys.2022.850707)
Supplement: Supplementary file 1 [file DataSheet1.pdf]

Guidelines for Reporting Reliability and Agreement Studies (GRRAS)

Section	Item	Page
Title and abstract	1. Identify in title or abstract that interrater/intrarater reliability or agreement was investigated.	P2
Introduction	2. Name and describe the diagnostic or measurement device of interest explicitly.	P5
	3. Specify the subject population of interest	P4
	4.Specify the rater population of interest (if applicable)	Not applicable
	5. Describe what is already known about reliability and agreement and provide a rationale for the study (if applicable)	P3-4
Methods	6. Explain how the sample size was chosen. State the determined number of raters, subjects/objects, and replicate observations	P4
	7. Describe the sampling method.	P4
	8. Describe the measurement/rating process	P5-6
	9. State whether measurements/ratings were conducted independently.	P5
	10. Describe the statistical analysis.	P6
Results	11.. State the number of rater and subjects/objects which were included and the number of replicate observations which were conducted	We described it in the methods section as follows:<br>Number of raters: P5<br>Number of subjects: P4.<br>Number of replicate observations: P5

	12. Describe the sample characteristics of raters and subjects.	Participants' characteristics: P4<br><br>Rater's characteristics: P5
	13. Report estimates of reliability and agreement including measures of statistical uncertainty	We reported all relevant data including measures of statistical uncertainty in tables. Including standard deviation, standard error of measurements.
Discussion	14. Describe the practical relevance of results	P7-8
Auxiliary Material	15. Provide detailed results if possible	The detailed results have presented in the tables.
